# Realtime optimization of multidimensional NMR spectroscopy on embedded sensing devices

**DOI:** 10.1038/s41598-019-53929-1

**Published:** 2019-11-25

**Authors:** Yiqiao Tang, Yi-Qiao Song

**Affiliations:** Schlumberger-Doll Research, Cambridge, MA 02139 USA

**Keywords:** Engineering, Mathematics and computing

## Abstract

The increasingly ubiquitous use of embedded devices calls for autonomous optimizations of sensor performance with meager computing resources. Due to the heavy computing needs, such optimization is rarely performed, and almost never carried out on-the-fly, resulting in a vast underutilization of deployed assets. Aiming at improving the measurement efficiency, we show an OED (Optimal Experimental Design) routine where quantities of interest of probable samples are partitioned into distinctive classes, with the corresponding sensor signals learned by supervised learning models. The trained models, digesting the compressed live data, are subsequently executed at the constrained device for continuous classification and optimization of measurements. We demonstrate the closed-loop method with multidimensional NMR (Nuclear Magnetic Resonance) relaxometry, an analytical technique seeing a substantial growth of field applications in recent years, on a wide range of complex fluids. The realtime portion of the procedure demands minimal computing load, and is ideally suited for instruments that are widely used in remote sensing and IoT networks.

## Introduction

NMR, considered as one of the most potent analytical methods, traditionally requires dedicated personnel and delicate equipment thanks to the use of superconducting magnets, sizable electronics, and intricate probe and antenna placements^1^. Only recently, owing to the advancement of permanent-magnet design^2^, integrated electronics^3,4^, and antenna miniaturization^5^, portable NMR systems^6^ have emerged as a viable surrogate. Thanks to the reductions in footprint, maintenance needs and price tag, the miniaturized sensor assemblies have extended their uses beyond conventional NMR laboratories to point-of-care medical diagnostics^7,8^, subterranean explorations^9^, flow metering^10^, fluid authentication^11^, and artefact preservation^12^. In those “field” applications, it is highly desirable that the machinery operates autonomously and self-optimizes based on properties of the samples under investigation.

The needs for optimizing NMR spectroscopy become more pressing when considering that the quantities of interest, such as relaxation times (*T*_1_ and *T*_2_), diffusion coefficient, J-coupling, and chemical shift oftentimes span a large numerical range up to several orders of magnitude^13^. Consequently, a fit-for-all-purpose pulse sequence (PS) often does not exist. Misuses of pulse sequence could result in either a prolonged experiment time or a loss of measurement accuracy. Previous efforts on measurement optimization have been focusing on samples of simple compositions (containing single or double fluid species) and/or 1D functional forms of forward models^14–16^.

Another challenge for autonomous optimization at the embedded sensing devices stems from the limited computing infrastructure, where microprocessors of merely tens of MHz CPU clock-rate and fast memories of tens of KB to a few MB are available^17^. The so-called “constrained devices” may connect to a gateway or cloud platform of much greater computing throughput, but often the connection is slow and intermittent^18,19^. In those scenarios, realtime optimizations need to be executed in its entirety at the sensory nodes of meager resources.

We wondered whether it would be possible to optimize multidimensional NMR relaxometry that measures NMR relaxation times^20^ of complex fluids, in realtime, on a mobile sensor generally regarded too “dumb” to perform such tasks. Instead of optimizing one sequence to all probable samples^15,16^, we utilized a suite of sequences that were individually optimized for samples with distinctive ranges of relaxation properties.

More specifically, we used the inversion-recovery-CPMG (IRCPMG) pulse sequence for *T*_1_ − *T*_2_ correlation spectroscopy. The forward model that describes the signal evolution as a function of experimental parameters is^21^:

1 $$S({\tau }_{1},{\tau }_{2})=\iint (1-\theta {e}^{-{\tau }_{1}/{T}_{1}}){e}^{-{\tau }_{2}/{T}_{2}}f({T}_{1},{T}_{2})d{T}_{1}d{T}_{2}+\epsilon ,$$

where $\epsilon$ is the experimental noise, *θ* is a calibration coefficient of the instrument, *τ*_1_ and *τ*_2_ are prescribed parameters that the spectrometer traverses through, with *S*(*τ*_1_, *τ*_2_) the corresponding recorded signals. Inversion methods^22^ are applied to obtain the sample {*T*_1_, *T*_2_} correlation spectrum, *f*(*T*_1_, *T*_2_). As real-life samples often have broadly distributed *f*(*T*_1_, *T*_2_), we made no assumptions other than *T*_1_ ≥ *T*_2_^23^ on the mathematical constructs of the spectra.

**Figure 1:**
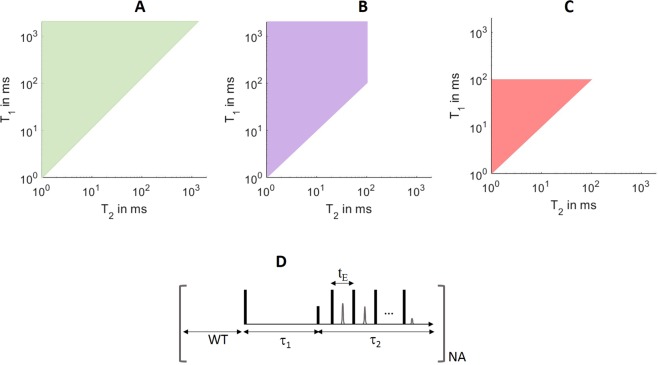
The three sample classes: (**A**) have *T*_1_ and *T*_2_ components that are longer than 0.1 s; (**B**) have components of large *T*_1_/*T*_2_ ratio; and (**C**) only have short *T*_1_ and *T*_2_ components up to 0.1 s. The IRCPMG pulse sequence is shown in (**D**), where *t*_*e*_ is echo spacing, *τ*_1_ is *T*_1_ encoding time that includes *N*_1_ steps of iteration, *τ*_2_ is echo time, WT is polarization time, and NA is the number of scans; the vertical bars represent RF pulses and the in-between triangles represent echo signals.

Three classes of fluids were considered in the sequence design. As shown in Fig. 1, class A contained components that were longer than 0.1 s in both *T*_1_ and *T*_2_ dimensions; class B embodied high *T*_1_/*T*_2_ ratios, where *T*_1_ had components longer than 0.1 s while *T*_2_ spanned [1 ms, 0.1 s]; and class C had relatively short *T*_1_ and *T*_2_ that each spanned [1 ms, 0.1 s]. Accordingly, Table 1 shows the optimal sequences for the respective fluid classes (i.e. sequence *α* for fluid A, *β* for B, and *γ* for C). In particular, both sequences *α* and *β* had *τ*_1_ up to 10 s, capable of measuring *T*_1_ up to 2 s, while sequence *γ* had the maximal *τ*_1_ of 1 s that sufficed to measure *T*_1_ up to 0.2 s. Meanwhile, sequence *α* had the maximal *τ*_2_ = *N*_*e*_ × *t*_*e*_ of 10 s, capable of measuring *T*_2_ up to 2 s, in contrast to sequences *β* and *γ* with the maximal *τ*_2_ of 0.6 s. The shorter echo spacing, *t*_*e*_, used in sequences *β* and *γ* than in sequence *α* could further help resolve fast *T*_2_ components (Fig. S1).

**Table 1:** The three optimal pulse sequences.

PS	*α*	*β*	*γ*
*t*_*e*_(*μ*s)	500	200	200
*N*_*e*_	20,000	3,000	3,000
*τ*_1,*min*_(ms)	1	1	0.5
*τ*_1,*max*_(ms)	10,000	10,000	1,000
WT (s)	10	10	5
*N*_1_	20	20	20
*t*_90_(*μ*s)	25	25	25
*t*_180_(*μ*s)	50	50	50
NA	4	4	4
runtime (s)	1608	964	472

PS is short for pulse sequence, *t*_*e*_ is echo spacing, *N*_*e*_ is the number of echoes, *τ*_1,*min*_ is the minimal *τ*_1_, *τ*_1,*max*_ is the maximal *τ*_1_, *N*_1_ is the number of *τ*_1_, WT is the polarization time, and NA is the number of acquisitions. *t*_90_ and *t*_180_ are respectively 90° and 180° pulse lengths that are experimentally determined, and runtime is the total experimental time.

**Figure 2:**
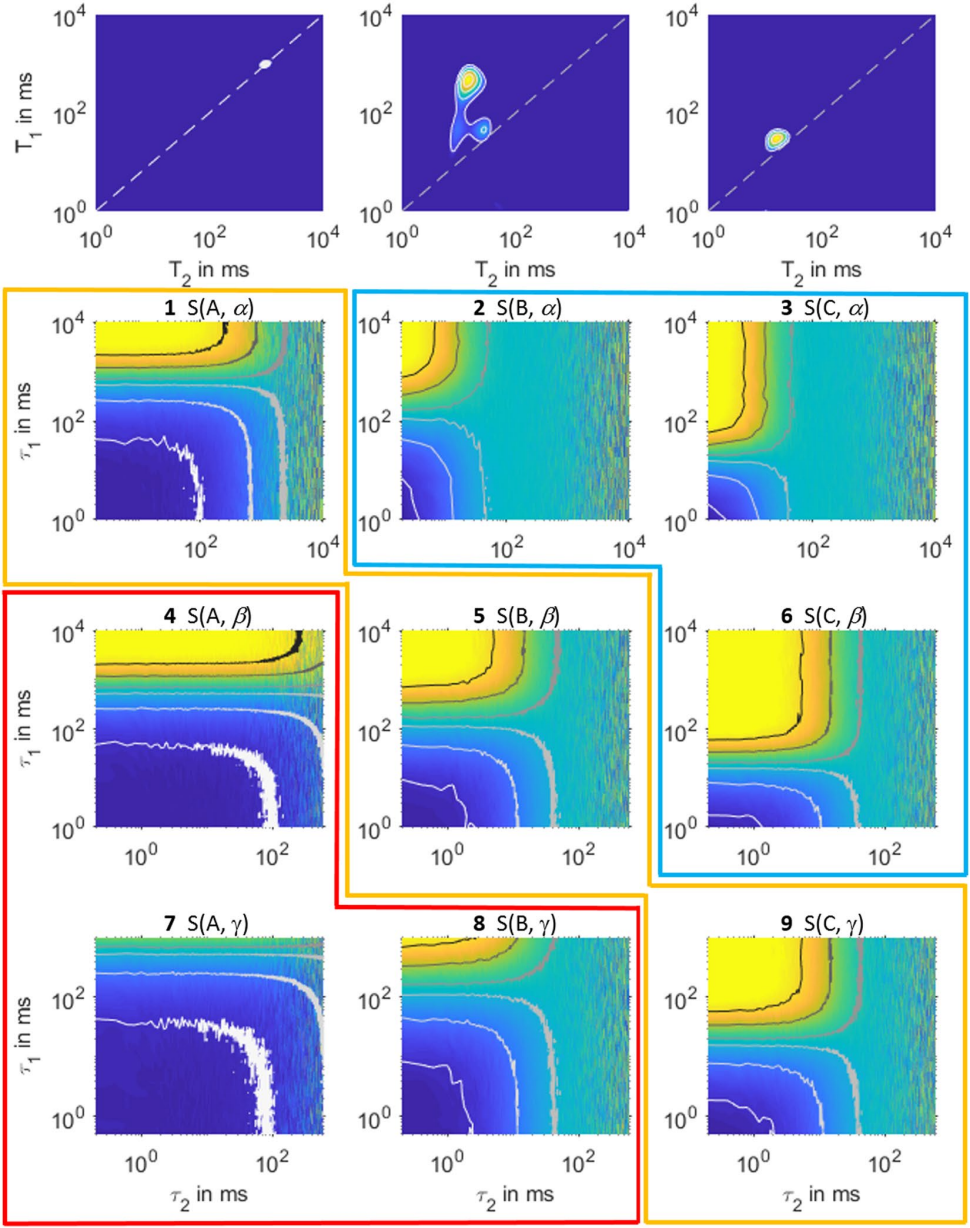
Top: *T*_1_ − *T*_2_ spectra of dodecane (fluid **A**), an emulsified fluid (fluid **B**), and glycerol (fluid **C**), acquired by sequences *α*, *β*, and *γ* respectively, and inverted using the Fast Laplace Inversion algorithm. Bottom: 9 log-log plots of time-domain patterns of NMR *T*_1_ − *T*_2_ measurements on the three fluids. Columns are by the fluid types, and rows by the applied sequences. Each pattern is normalized by the first acquisition point of the longest *τ*_1_ time at the top left corner, and contour lines, from bottom to top of darkening colors, represent −0.9, −0.5, −0.1, 0.1, 0.5, and 0.9. Measurements are optimal within the yellow envelope, inefficient within the blue, and erroneous within the red.

Ideally, sequences shall always be applied to the intended samples under study; but in continuous measurements on samples of changing properties, occasions do arise in which a sequence is suboptimally applied. As sensors generally couldn’t foresee temporal progression of sample properties, any combinations of fluid class and pulse sequence are practically probable. Here we show applications of each sequence to three exemplary fluids, namely dodecane (fluid A), emulsified fluid (fluid B), and glycerol (fluid C), in Fig. 2. In the 3 by 3 panels, the diagonal time-domain images were acquired by the respective optimal sequences, while the measurements that corresponded to the off-diagonal ones were either inefficient or erroneous (Fig. S1). The key challenge was to discern the fluid class from live time-domain images, regardless of the sequences in use, and apply the intended one in the subsequent runs. In practice, we used three trained ECOC (error-correcting output codes) learners^24^, a class of supervised learning models, for the realtime multiclass classification and inference task.

Supervised learning requires large quantities of labeled datasets for model training. The training sets can consist of either prior measurements on samples of known properties or forward-modeled simulations. We opted for the later approach thanks to the well-defined functional form of Eq. 1. Specifically, we approximated probable *T*_1_ − *T*_2_ distributions by a large ensemble of synthetic distributions, each consisting of three components of randomly generated $\{{\tilde{T}}_{1},{\tilde{T}}_{2}\}$ pairs. Since the measurement volume was a constant and filled with fluids of similar proton density, we assigned each component by a randomly-generated weighting coefficient, $\tilde{\mu }$, that sums to unity. The time-domain data, *S*_*T*_, for model training were generated as:

2 $${S}_{T}({\tau }_{1},{N}_{e},{t}_{e})=\mathop{\sum }\limits_{n=1}^{n=3}\,{\tilde{\mu }}_{n}(1-\theta {e}^{-{\tau }_{1}/{\tilde{T}}_{1,n}}){e}^{-{N}_{e}\cdot {t}_{e}/{\tilde{T}}_{2,n}}+{\epsilon }_{T},$$

where ${\sum }_{n=1}^{n=3}{\tilde{\mu }}_{n}=1$. After calibrating the experimental setup, we set *θ* to 1.85 and ${\epsilon }_{T}$ to a Gaussian noise with zero mean and 0.012 standard deviation.

To generate $\{{\tilde{T}}_{1},{\tilde{T}}_{2}\}$ pairs, we stochastically sampled in a two-dimensional space, where each dimension consisted of 100 logarithmically distributed numbers from 1 ms to 2 s and all pairs satisfied the relation ${\tilde{T}}_{1}\ge {\tilde{T}}_{2}$. In total, 30,000 ${\tilde{T}}_{1}-{\tilde{T}}_{2}$ distributions were created. Subsequently, we sifted the sampled distributions, one by one, through a set of classification criteria, and labeled them accordingly (Fig. S3). A given distribution was labeled class A if the longest ${\tilde{T}}_{2}$ > 0.1 s and its associated weighting coefficient ≥0.05, labeled class B if the longest ${\tilde{T}}_{2}$ < 0.1 s, its associated ${\tilde{T}}_{1}$ > 0.15 s, and its associated weighting coefficient ≥0.05, and labeled C if the longest ${\tilde{T}}_{1}$ < 0.1 s. As a result, 11,663 were assigned to class A, 11,835 to B, and 1449 to C.

To further reduce the size of training datasets, we exploited the separable structures of the functional form, and applied singular value decomposition (SVD) on *T*_1_ and *T*_2_ kernels independently^22^. Consequently, for any given sample, the size of compressed datasets was 1.57 KB when acquired by sequence *α*, 1.34 KB when acquired by *β*, and 1.25 KB when acquired by *γ*. As elaborated in the supplementary information, a near 1000-fold reduction in memory usage was achieved with the SVD compression.

Subsequently, we trained three ECOC classifiers, an ensemble method for multiclass classification problem^24^; each classifier is used while running the corresponding pulse sequence. The ECOC models encoded the binary classification results from three linear support vector machines (SVM) into a coding design matrix^25^, using the “one-versus-one” strategy that distinguished a pair of labeled time-domain patterns in the training set while ignoring the third fluid class. For sequences *α*, *β*, and *γ*, the ECOC classifiers had the respective size of 4.7, 4.1 and 3.8 KB. In total, less than 13 KB of fast memory was required to store the models.

In the inference stage of classifying a new 2D NMR dataset, we utilized the loss-weighted decoding scheme^26^ to aggregate predictions of the binary learners, in which the weighted “hinge” error functions^25^ over all binary losses were minimized. After running a pulse sequence, the number of floating-point calculations for classifying the generated data, after normalization and SVD compression, is fewer than 700. More details of the model training, validation and inference can be found in the supplementary information.

**Figure 3:**
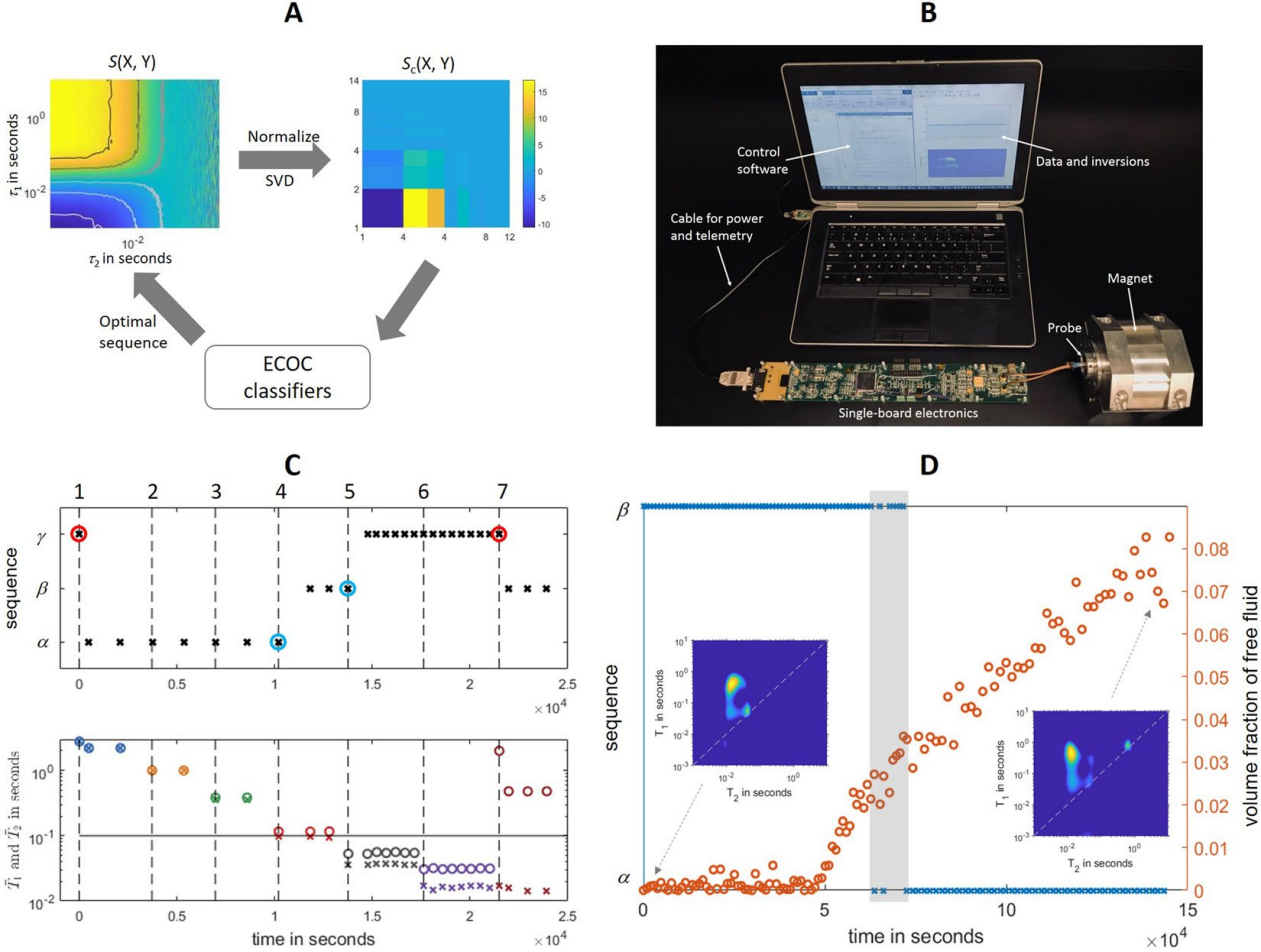
Realtime optimizations of continuous NMR *T*_1_ − *T*_2_ experiments with ECOC classifiers. (**A**) The optimization workflow where *S*(X,Y) is the raw time-domain pattern from fluid of class X acquired by sequence Y and *S*_*c*_(X,Y) is the pattern after normalization and SVD compression; (**B**) A photo of the miniaturized NMR system; (**C**) A series of NMR experiments on sequentially displaced fluid samples. Sample 1 to 6 are mixtures of glycerol and water with different volume ratios: 0:100 (1), 10:90 (2), 20:80 (3), 50:50 (4), 70:30 (5), and 100:0 (6); sample 7 is a well-gelled emulsified fluid. The vertical dashed lines mark the time stamps of sample injection. In the upper panel, the crosses signify applied sequences, with the red and blue circles highlighting inaccurate and inefficient measurements, respectively. In the lower panel, the circles and crosses signify calculated means of *T*_1_ and *T*_2_ times from the obtained 2D datasest; (**D**) Continuous NMR measurements on an emulsified fluid under static conditions, where y axis on the left indicates the sequence in use, and on the right indicates the calculated volume fraction of bulk base oil (any components with *T*_2_ > 0.1 s). The light gray band highlights the transition of optimal sequences. The inset shows the *T*_1_ − *T*_2_ correlation spectra of the fluid at the two marked time stamps.

We performed realtime optimizations of continuous NMR experiments with the trained ECOC classifiers, as illustrated in Fig. 3. The mobile NMR sensor^27^, shown in Fig. 3B, was miniaturized largely due to the use of an NMR ASIC (Application Specific Integrated Circuit)^3^. The NMR probe, embodied in a Halbach-array magnet, was made of a solenoid coil wound around a polymer capillary, interrogating fluid samples of 17 *μ*L in volume. During operation, the spectrometer executed a selected pulse sequence that was downloaded from the laptop, on which the acquired data were input to ECOC classifiers for realtime inference.

Figure 3C shows a series of experiments on sequentially displaced samples. The samples were six water/glycerol mixes of varying volume fractions and one emulsified fluid. As the volume fraction of glycerol increases, the relaxation times of the mixtures shorten from *T*_2_ = *T*_1_ = 2 s of pure water to *T*_2_ = *T*_1_/2 = 15 ms of pure glycerol; meanwhile, the *T*_1_/*T*_2_ ratio also inflates thanks to the elevated fluid viscosity. We started with sample 1 of pure water while applying sequence *γ*; the classifier correctly identified the fluid as type A and instructed to use sequence *α* for the subsequent run. Thereafter, sequence *α* was optimally applied for samples 1, 2 and 3. Sample 4 had *T*_1_/*T*_2_ slightly above 1 with *T*_2_ = 0.1 s. Consequently, the optimization routine signified the sample as type B. Sequence *β* was properly applied till sample 5 was loaded, which was classified as fluid C. Subsequently, sequence *γ* was applied on sample 5 and 6 of rather short relaxation times. Finally, we displaced glycerol by a well-gelled emulsion sample, which the ECOC classifier correctly deduced as a class B fluid; the spectrometer subsequently applied sequence *β* for the rest of the experiments.

In addition to physical displacement, a given sample could also evolve over time. For example, the emulsion fluids, which are multiphasic mixes of oil, brine, organoclays, and naturally-mined barite particles, could experience phase separation under static conditions. As the emulsion collapsed and solid particles sedimented, the emancipated oil exhibited a characteristic *T*_2_ time much longer than the original fluid, calling for a different optimal sequence.

Experimentally, we performed the optimization routine for continuously monitoring an emulsion sample under static conditions. As shown in Fig. 3D, the initial well-gelled emulsion fell in the class B fluid, to which sequence *β* was optimally applied. At about 50,000 s after the experiment commenced, signals of bulk oil started to appear with a *T*_2_ at ca. 0.5 s. As volume fraction of free oil increased, the fluid gradually morphed to class A, with the corresponding optimal sequence *α*. Notably, A transition window presented at ca. 60,000 s, where the free fluid content was still marginal while the inference results hinged partially on noise realizations of each measurement.

In practice, it is important to ensure that each sequence has sensitivities over the entire numerical domain of *T*_1_ − *T*_2_ spectra under consideration. Failure to meet the requirement could cause misclassification and thereby erroneous results. For example, the maximum *τ*_1_ in sequence *γ*, which is optimized for samples of fast relaxation times, should be designed so that the signal decays significantly with the maximal *T*_1_. Mathematically, it should satisfy 1 − *exp*(−*τ*_1,*max*_/*T*_1,*max*_) ≫ *σ*, where *σ*^2^ is the variance of the Gaussian noise. The relation is indeed held in the work, as *τ*_1,*max*_ = 1 s for sequence *γ*, *T*_1,*max*_ = 2 s, and *σ*^2^ = 0.012^2^.

Although we focus on relaxometry, the method can be extended to other types of NMR measurements of increasing complexities, such as multidimensional spectroscopy and MRI, at the core of which are forward models of similar mathematical constructs (exponential, sine and cosine functions). In conjunction with minimal requirements on computing resources, the demonstrated approach may further NMR methods to a substantially broadened usage in a wide range of field applications.

## Supplementary information

Supplementary information

## References

[1] Günther, H. *NMR spectroscopy: basic principles, concepts and applications in chemistry* (John Wiley & Sons, 2013).

[2] Danieli E, Perlo J, Blümich B, Casanova F (2010). Small magnets for portable NMR spectrometers. Angew. Chem. Int. Ed.

[3] Ha D, Paulsen JL, Sun N, Song Y-Q, Ham D (2014). Scalable NMR spectroscopy with semiconductor chips. Proceedings of the National Academy of Sciences.

[4] Huber S (2019). Multichannel digital heteronuclear magnetic resonance biosensor. Biosensors and Bioelectronics.

[5] Wensink H (2005). Measuring reaction kinetics in a lab-on-a-chip by microcoil NMR. Lab on a Chip.

[6] Zalesskiy SS, Danieli E, Blumich B, Ananikov VP (2014). Miniaturization of NMR systems: Desktop spectrometers, microcoil spectroscopy, and “NMR on a chip” for chemistry, biochemistry, and industry. Chemical reviews.

[7] Colucci LA (2019). Fluid assessment in dialysis patients by point-of-care magnetic relaxometry. Science Translational Medicine.

[8] Jeong S (2017). Real-time quantitative analysis of metabolic flux in live cells using a hyperpolarized micromagnetic resonance spectrometer. Science Advances.

[9] Kleinberg, R. *et al*. Deep sea NMR: Methane hydrate growth habit in porous media and its relationship to hydraulic permeability, deposit accumulation, and submarine slope stability. *Journal of Geophysical Research: Solid Earth* **108** (2003).

[10] Fridjonsson EO, Stanwix PL, Johns ML (2014). Earth’s field NMR flow meter: Preliminary quantitative measurements. Journal of Magnetic Resonance.

[11] Pinter M, Harter T, McCarthy M, Augustine M (2014). Towards Using NMR to Screen for Spoiled Tomatoes Stored in 1,000L, Aseptically Sealed, Metal-Lined Totes. Sensors.

[12] Blumich B (2010). Noninvasive testing of art and cultural heritage by mobile NMR. Accounts of Chemical Research.

[13] Levitt, M. H. *Spin dynamics: basics of nuclear magnetic resonance* (John Wiley & Sons, 2001).

[14] Song Y-Q, Tang Y, Hürlimann M, Cory D (2018). Real-time optimization of nuclear magnetic resonance experiments. Journal of Magnetic Resonance.

[15] Jones J, Hodgkinson P, Barker A, Hore P (1996). Optimal sampling strategies for the measurement of spin–spin relaxation times. Journal of Magnetic Resonance, Series B.

[16] Reci A, Ainte M, Sederman AJ, Mantle MD, Gladden LF (2019). Optimising sampling patterns for bi-exponentially decaying signals. Magnetic resonance imaging.

[17] Chiang M, Zhang T (2016). Fog and iot: An overview of research opportunities. IEEE Internet of Things. Journal.

[18] Tubel, P., Bergeron, C. & Bell, S. Mud pulser telemetry system for down hole measurement-while-drilling. In *Instrumentation and Measurement Technology Conference*, 1992. IMTC’92., 9th IEEE, 219–223 (IEEE, 1992).

[19] Jarrot A, Gelman A, Kusuma J (2018). Wireless digital communication technologies for drilling: Communication in the bits/s regime. IEEE Signal Processing Magazine.

[20] Bloembergen N, Purcell EM, Pound RV (1948). Relaxation effects in nuclear magnetic resonance absorption. Physical review.

[21] Song Y-Q (2002). T_1 _− T_2_ correlation spectra obtained using a fast two-dimensional Laplace inversion. Journal of Magnetic Resonance.

[22] Venkataramanan L, Song Y-Q, Hurlimann MD (2002). Solving Fredholm integrals of the first kind with tensor product structure in 2 and 2.5 dimensions. IEEE Transactions on Signal Processing.

[23] Traficante DD (1991). Relaxation. Can T_2_ be longer than T_1_?. Concepts in Magnetic Resonance.

[24] Dietterich TG, Bakiri G (1994). Solving multiclass learning problems via error-correcting output codes. Journal of artificial intelligence research.

[25] Bishop, C. M. *Pattern recognition and machine learning* (Springer, 2006).

[26] Escalera S, Pujol O, Radeva P (2010). On the decoding process in ternary error-correcting output codes. IEEE transactions on pattern analysis and machine intelligence.

[27] Tang Y, McCowan D, Song Y-Q (2019). A miniaturized spectrometer for NMR relaxometry under extreme conditions. Scientific reports.

