# Supplementary material for: Realtime optimization of multidimensional NMR spectroscopy on embedded sensing devices
Source: Sci Rep. 2019 Nov 25;9:17486. doi: 10.1038/s41598-019-53929-1 (PMC6877539; doi:10.1038/s41598-019-53929-1)
Supplement: Supplementary file 1 — Supplementary information [file 41598_2019_53929_MOESM1_ESM.pdf]

Supplementary materials for "Realtime optimization of multidimensional NMR spectroscopy on embedded sensing devices"

Yiqiao Tang,[1*] Yi-Qiao Song,[1]

[1]Schlumberger-Doll Research, Cambridge, MA 02139

[*]To whom correspondence should be addressed; E-mail: ytang12@slb.com.

Materials and Methods

Materials

The emulsified fluid used in the study was an invert emulsion (*1*) that consisted of oil (50%), brine (20%), naturally-mined barite particles (20%), and a multitude of polymer/clay additives (10%). The numbers in the brackets are volume fractions of each component. When well gelled, the sample presented short T_2 relaxation times (<50 ms) and large T_1/T_2 ratio (>100). We attribute these spectra features to a combination of restricted molecular motions and proton-electron interactions with ferromagnetic species (*2*).

All other chemicals used in the study were procured from Sigma Aldrich.

2D inversions of time-domain signals

In Fig. S1, we show 2D inversion results on the time-domain datasets, displayed in Fig. 2 of the main article, of the three exemplary fluids.

Inaccurate measurements may result in erroneous estimates of T_2 (sequence β on dodecane), or T_1 (sequence γ on emulsified fluid), or both T_1 and T_2 (sequence γ on dodecane). On the other hand, inefficient measurements result in prolonged experiments (for example, with similar data quality, runtime of applying sequence α on the emulsion and glycerol is ca. $2\times$ and $4\times$ of runtime of their respective optimal sequence). Finally, we note that short t_e, when used properly, helps resolve spectral features. This is manifested by the higher T_2 resolutions of the emulsified fluid and glycerol with sequence β and γ than with sequence α.

In Fig. S2, we show $T_1 - T_2$ spectra of the six water/glycerol mixes.

Sampled $\tilde{T}_1 - \tilde{T}_2$ distributions for model training

In Fig. S3, we show the 24,918 sampled $\tilde{T}_1 - \tilde{T}_2$ distributions for model training, of which 11,663 were assigned to class A fluid, 11,835 to class B fluid, and 1449 to class C fluid.

Singular Value Decomposition (SVD)

SVD is a powerful compression technique for reducing data dimensionality while preserving the essential feature information (*3, 4*). We first denote $k_1 = (1 - \theta e^{-\tau_1/T_1})$ and $k_2 = e^{-\tau_2/T_2}$, then Eq. 1 can be written in a matrix notation:

$$S = K_1 F K_2' + E, \tag{S1}$$

where matrices K_1, K_2 and F are the respectively discretized versions of k_1, k_2 and f. Subsequently, we factorize both matrices $K_{1,2}$ into their singular vectors, $U_{1,2}$ and $V_{1,2}^T$, mediated by $\Sigma_{1,2}$ composed of $K_{1,2}$'s singular values through $K_{1,2} = U_{1,2}\Sigma_{1,2}V_{1,2}^T$. Combining the SVDs with Eq. S1, one obtains:

$$\tilde{S} = \tilde{K_1} F \tilde{K_2}' \tag{S2}$$

where $\tilde{S} = U_1' S U_2$, $\tilde{K}_1 = \Sigma_1 V_1'$, and $\tilde{K}_2 = \Sigma_2 V_2'$. Here we use the fact that both U_1 and U_2 are unitary matrices. In practice, we use truncated SVD to the tth order with the condition number of 10,000, i.e. $s_0/s_t \leq 10,000$ but $s_0/s_{t+1} > 10,000$, where s_i are the singular values of descending order.

Eq. S2 describes data compression through applying SVD. For example, the raw time-domain data, S, for sequence γ is a 2D matrix of 20 $\times$ 20,000; t_1 and t_2 for the two kernels are both 14. U_1 and U_2 are matrices of respective dimension 14 $\times$ 20 and 14 $\times$ 20,000. As a result, $\tilde{S}$ has a dimension of 14 $\times$ 14. In Fig. S4, we plotted the means of SVD-compressed data of all the training sets.

U_1 and U_2 for all sequences will need to be stored on the embedded device for realtime data compression. U_2 can be a large matrix, especially with a long CPMG train. In the current work, size of U_2 is 2.24 MB, 386 KB, and 312 KB for sequence α, β, γ. In practice, other techniques, such as window-sum compression (*5*), may be used to further reduce the matrix size.

Support Vector Machine (SVM) models

Linear SVMs are a class of supervised learning models for binary classification tasks (*6*). Mathematically, for data entries consisting of N points, a trained SVM classifier contains N weights (W_i, where $i = 1, 2, \dots N$) and one bias (B). The numerical values of the weights and the bias are determined while training the model.

When classify a new dataset, $\mathbf{X}$, the classifier calculates a score of the learner, $s = \mathbf{X} \cdot \mathbf{W} + B$. Depending on the sign of the result, it is assigned to one class (if sign($\mathbf{X} \cdot \mathbf{W} + B$) >0) or the other (if sign($\mathbf{X} \cdot \mathbf{W} + B$) <0). For the three sequences, the SVD-compressed datasets have the respective length of 196, 168 and 156. Consequently, the corresponding SVMs have the size of 1.58 KB, 1.35 KB, and 1.26 KB.

Error-correcting Output Codes (ECOC) models

For multiclass classification, an ECOC model reduces the original task into a set of binary problems, which is separately dealt with using SVM models.

In the training stage, we use the "one-vs-one" strategy to direct which classes are trained by each binary learner. The utilized coding design, $\{m_{ij}\}$ (where $i =$ and $j = 1, 2, 3$), is:

	SVM_1	SVM_2	SVM_3
class A	1	1	0
class B	-1	0	1
class C	0	-1	-1

Table 1: Code design of the "one-vs-one" strategy

SVM_1 trains on labeled datasets in class A or class B, and treats class A as the positive and lass B as the negative. The other learners are trained similarly. Since each ECOC model includes three SVM learners, its size correspondingly triples.

In the inference stage, the algorithm assigns a new dataset to one of the three classes, $\hat{i}$, that minimizes the aggregation of hinge losses (*7*):

$$\hat{i} = \underset{i}{\operatorname{argmin}} \sum_{j=1}^{3} \max(0, 1 - m_{ij} \cdot s_j), \tag{S3}$$

Finally, we validated the trained ECOC models with 3000 randomly generated $\tilde{T}_1 - \tilde{T}_2$ distributions. The error rate was below 0.1% for all three sequences.

References

1. A. D. Patel, Invert emulsion fluids suitable for drilling (1999). US Patent 5,905,061.

2. L. F. Gladden, J. Mitchell, Measuring adsorption, diffusion and flow in chemical engineering: applications of magnetic resonance to porous media. *New Journal of Physics* **13**, 035001 (2011).

3. L. Venkataramanan, Y.-Q. Song, M. D. Hurlimann, Solving Fredholm integrals of the first kind with tensor product structure in 2 and 2.5 dimensions. *IEEE Transactions on Signal Processing* **50**, 1017–1026 (2002).

4. Y.-Q. Song, L. Venkataramanan, M. D. Hurlimann, M. Flaum, P. Frulla, C. Straley, T_1-T_2 correlation spectra obtained using a fast two-dimensional Laplace inversion. *J. Magn. Reson.* **154**, 261-268 (2002).

5. N. J. Heaton, Multi-measurement NMR analysis based on maximum entropy (2005). US Patent 6,960,913.

6. C. M. Bishop, *Pattern recognition and machine learning* (springer, 2006).

7. K.-B. Duan, S. S. Keerthi, *International workshop on multiple classifier systems* (Springer, 2005), pp. 278–285.

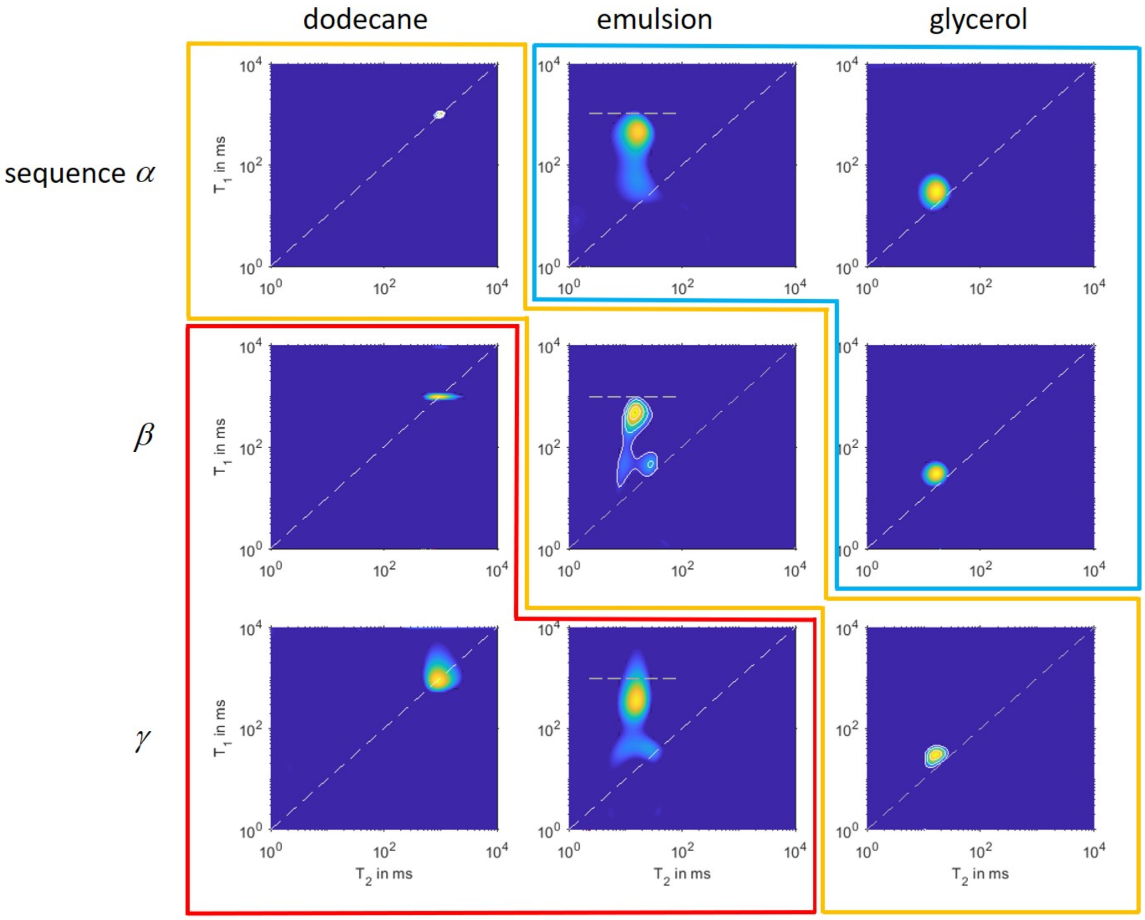

Fig. S1. $T_1 - T_2$ spectra by inverting the time-domain datasets shown in Fig. 2. The horizontal dashed lines in the middle column signify $T_1 = 1$ s.

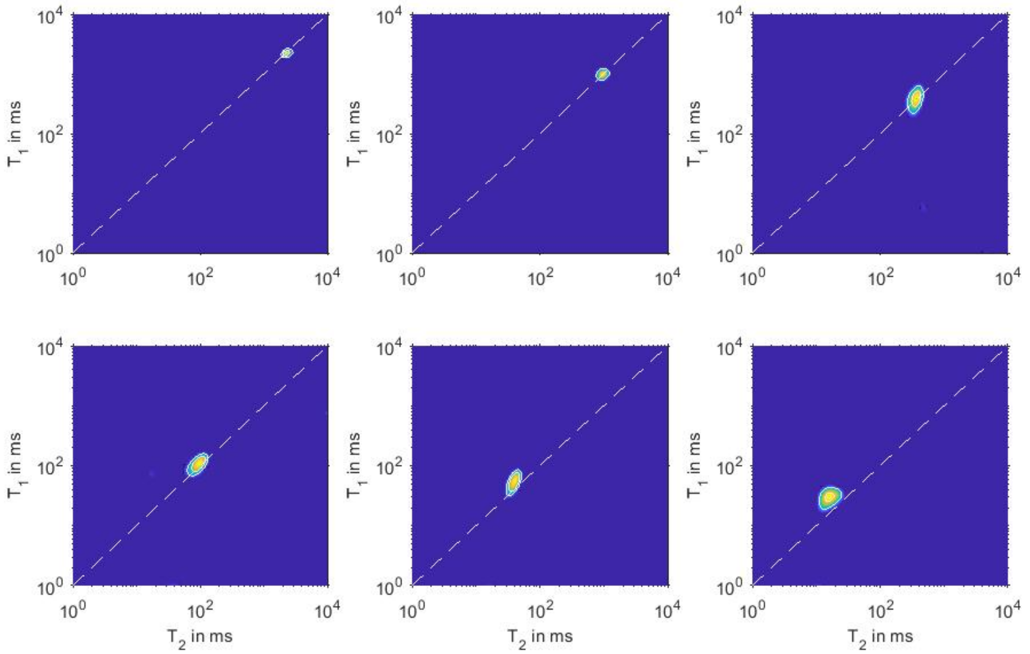

Fig. S2. $T_1 - T_2$ spectra of the six water/glycerol mixes, shown in Fig. 3C.

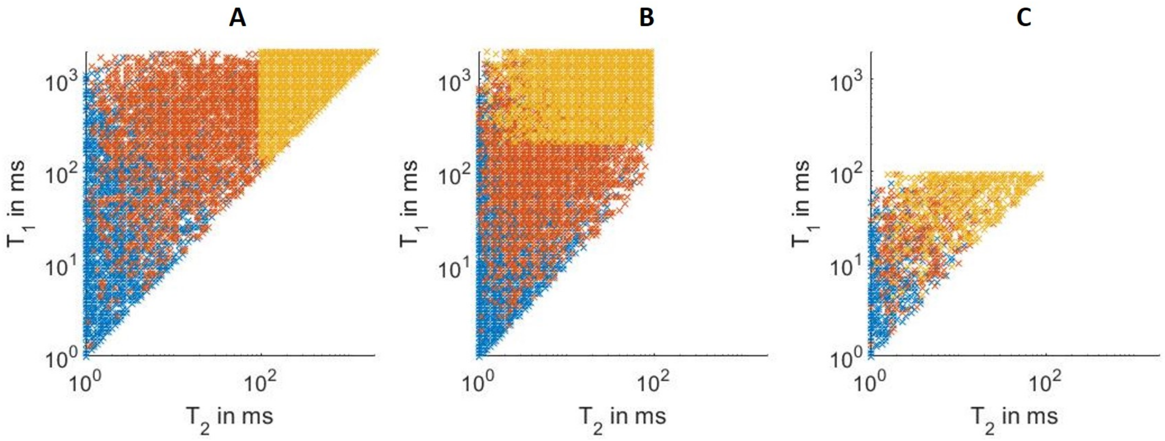

Fig. S3. The sampled $\tilde{T}_1 - \tilde{T}_2$ distributions labeled by the fluid classes. The three colors show the respective components, in yellow, red, blue of descending $\tilde{T}_2$'s. A given distribution is assigned to class A if the longest $\tilde{T}_2 > 0.1$ s with the associated weighting coefficient ≥ 0.05, to class B if the longest $\tilde{T}_2 < 0.1$ s, the longest $\tilde{T}_1 > 0.15$ s with the weighting coefficient $\geq$ 0.05, and to class C if the longest $\tilde{T}_1 < 0.1$ s.

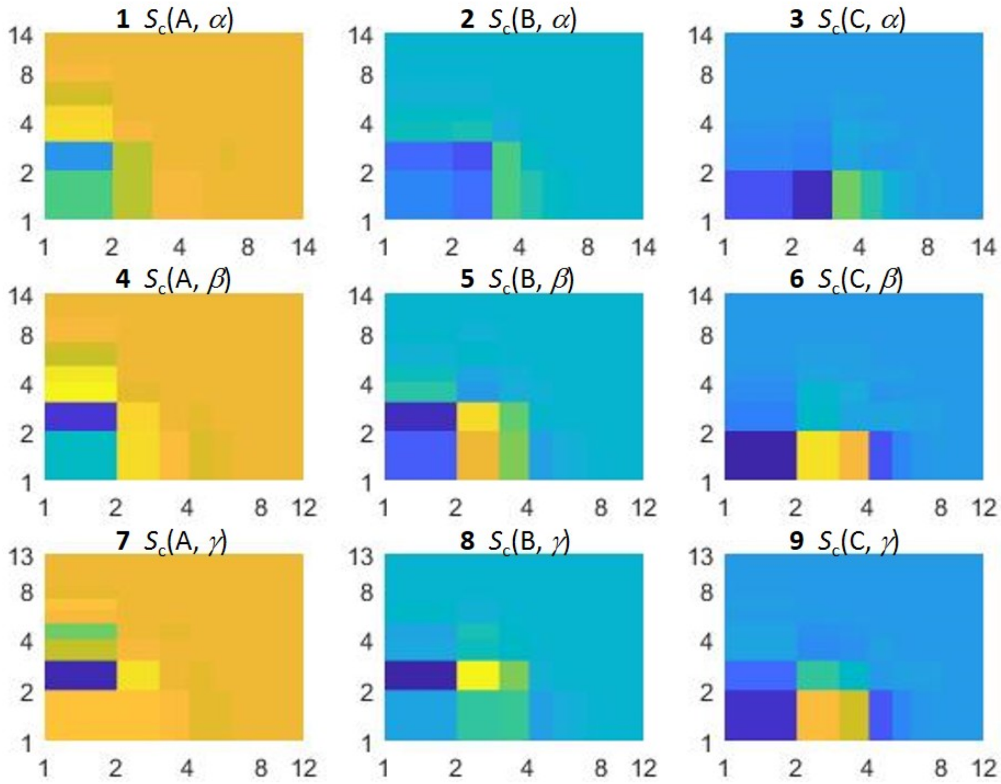

Fig. S4. means of SVD-compressed imagies of all training datasets.
